# Root Damage under Alkaline Stress Is Associated with Reactive Oxygen Species Accumulation in Rice (*Oryza sativa* L.)

**DOI:** 10.3389/fpls.2017.01580

**Published:** 2017-09-08

**Authors:** Hui Zhang, Xiao-Long Liu, Rui-Xue Zhang, Hai-Yan Yuan, Ming-Ming Wang, Hao-Yu Yang, Hong-Yuan Ma, Duo Liu, Chang-Jie Jiang, Zheng-Wei Liang

**Affiliations:** ^1^Northeast Institute of Geography and Agroecology, Chinese Academy of Sciences Changchun, China; ^2^Da’an Sodic Land Experiment Station, Chinese Academy of Sciences, Da’an Jilin, China; ^3^College of Resources and Environment, University of Chinese Academy of Sciences Beijing, China; ^4^Institute of Agrobiological Sciences, National Agriculture and Food Research Organization Tsukuba, Japan

**Keywords:** rice (*Oryza sativa* L.), alkaline stress, root damage, reactive oxygen species (ROS), seedling growth

## Abstract

Alkaline stress (high pH) severely damages root cells, and consequently, inhibits rice (*Oryza sativa* L.) seedling growth. In this study, we demonstrate the accumulation of reactive oxygen species (ROS) in root cells under alkaline stress. Seedlings of two rice cultivars with different alkaline tolerances, ‘Dongdao-4’ (moderately alkaline-tolerant) and ‘Jiudao-51’ (alkaline-sensitive), were subjected to alkaline stress simulated by 15 mM sodium carbonate (Na_2_CO_3_). Alkaline stress greatly reduced seedling survival rate, shoot and root growth, and root vigor. Moreover, severe root cell damage was observed under alkaline stress, as shown by increased membrane injury, malondialdehyde accumulation, and Evan’s Blue staining. The expression of the cell death-related genes *OsKOD1*, *OsHsr203j*, *OsCP1*, and *OsNAC4* was consistently upregulated, while that of a cell death-suppressor gene, *OsBI1*, was downregulated. Analysis of the ROS contents revealed that alkaline stress induced a marked accumulation of superoxide anions ($O_{2}^{•-}$) and hydrogen peroxide (H_2_O_2_) in rice roots. The application of procyanidins (a potent antioxidant) to rice seedlings 24 h prior to alkaline treatment significantly alleviated alkalinity-induced root damage and promoted seedling growth inhibition, which were concomitant with reduced ROS accumulation. These results suggest that root cell damage, and consequently growth inhibition, of rice seedlings under alkaline stress is closely associated with ROS accumulation. The antioxidant activity of superoxide dismutase, catalase, peroxidase, and ascorbate peroxidase increased under alkaline stress in the roots, probably in response to the cellular damage induced by oxidative stress. However, this response mechanism may be overwhelmed by the excess ROS accumulation observed under stress, resulting in oxidative damage to root cells. Our findings provide physiological insights into the molecular mechanisms of alkalinity-induced damage to root cells, and will contribute to the improvement of alkaline stress tolerance in rice plants.

## Introduction

Saline–alkaline (SA) stress due to soil salinization and/or alkalization is a major constraint to crop production worldwide. It is estimated that, globally, about 8.3 × 10^8^ ha of land are affected by salt, over half of which are alkaline (Martinez-Beltran and Manzur, 2005). SA stress is characterized by an inordinately high alkalinity (high pH) in addition to high salinity; the pH of SA soil can range from 8.5 to 11 (Abrol et al., 1988; Qadir et al., 2006, 2007; Hossner, 2008; Wang et al., 2009; Amini et al., 2016). Plants growing in SA soil suffer from high saline toxicity and damage induced by alkaline conditions. To date, numerous studies have focused on the molecular mechanisms underlying plant responses to salt stress, leading to the development of a number of salt-tolerant crop plants (Singh et al., 2008; Amudha and Balasubramani, 2011; Hanin et al., 2016; Gilliham et al., 2017). In contrast, much less attention has been directed toward alkaline stress, which is becoming an increasingly important stress factor that is considerably more damaging to plants than any single neutral saline stress (Qadir et al., 2001; Hartung et al., 2002; Yang et al., 2008; Islam et al., 2011; Chen et al., 2012; Paz et al., 2012; Radi et al., 2012; Shasha, 2012; Lv et al., 2013). It is essential to first understand how plants respond and adapt to alkaline stress, in order to efficiently improve the alkaline tolerance of crop plants.

Plants frequently encounter one or more abiotic stressors in their natural habitats, such as drought, salinity, and extreme temperature. In response to these stressors, plants generate and accumulate significant levels of reactive oxygen species (ROS), such as superoxide anions ($O_{2}^{•-}$), hydrogen peroxide (H_2_O_2_), hydroxyl radicals (•OH), and singlet oxygen (^1^O_2_) (Choudhury et al., 2017). These ROS molecules play important signaling roles in response to a wide range of developmental and environmental stimuli (Choudhury et al., 2017; Mittler, 2017). ROS production, and the associated redox regulation, is one of the most common plant responses to stress, and represents a point of integration for various signaling pathways (Sewelam et al., 2016). Conversely, high levels of ROS are harmful to living cells; excess ROS accumulation induces oxidative damage to cellular membranes (lipid peroxidation), proteins, RNA, and DNA, resulting in irreversible cellular damage, and even cell death (Sewelam et al., 2016; Choudhury et al., 2017; Kao, 2017). Plant cells are able to neutralize ROS through an elaborate antioxidant defense system consisting of ROS-scavenging enzymes and antioxidants (Sewelam et al., 2016; Choudhury et al., 2017; Kao, 2017). ROS-scavenging enzymes include superoxide dismutase (SOD), catalase (CAT), peroxidase (POD), and ascorbate peroxidase (APX), and endogenous antioxidants, including ascorbic acid (AsA) and reduced glutathione (GSH). It appears that a limited and controlled rise in ROS is required for a number of beneficial cellular responses to stress, while prolonged ROS production may exceed the plant’s antioxidant defense capability and result in oxidative damage. The balance between ROS production and ROS elimination can be severely disturbed by various stressors, resulting in excess accumulation of intracellular ROS, and consequently, damage to plant cells (Caverzan et al., 2016).

In rice (*Oryza sativa* L.), a number of studies have reported H_2_O_2_ accumulation in response to salt treatment, and have shown that oxidative stress due to high salinity is a major cause of cellular damage and plant growth inhibition (Nor’aini et al., 1997; Lee et al., 2001; Lin and Kao, 2001; Tsai et al., 2005; Hong et al., 2009; Yamane et al., 2012; Wei et al., 2013; Hazman et al., 2015; Kao, 2017). Reduced root growth in response to high salinity in rice seedlings (Lin and Kao, 2001; Lv et al., 2013) is closely correlated with increased H_2_O_2_ levels (Lin and Kao, 2001; Tsai et al., 2005; Hong et al., 2009). Exogenous H_2_O_2_ treatment has been consistently shown to inhibit the root growth of rice seedlings (Lin and Kao, 2001). In response to salt stress, rice plants activate antioxidant defense systems by upregulating the expression and activity of ROS-scavenging enzymes (Lin and Kao, 2001; Tsai et al., 2004, 2005; Moradi and Ismail, 2007; Hong et al., 2009). The transgenic overexpression of antioxidant enzyme genes enhances ROS-scavenging activity and salt tolerance in rice plants (Tanaka et al., 1999; Zhao and Zhang, 2006; Motohashi et al., 2007; Kumar et al., 2014). In addition, a rice mutant line *coleoptile photomorphogenesis 2* (*cpm2*), which is defective in the gene encoding allene oxide cyclase (*OsAOC*), exhibits enhanced salt tolerance with a concomitant decrease in ROS accumulation (Hazman et al., 2015). These data demonstrate a strong correlation between salt tolerance and ROS-scavenging capabilities in rice plants (Kao, 2017).

High pH conditions impose severe damage stress on rice seedling growth by causing serious root cell injury and death, which, consequently, results in whole-plant wilting and even death (Lv et al., 2013; Guo et al., 2014; Wei et al., 2015). However, the molecular mechanism responsible for root damage by alkaline stress remains largely unknown. Recently, Guo et al. (2014) identified a rice mutant, *alkaline tolerance 1* (*alt1*), with enhanced tolerance to alkaline stress (Guo et al., 2014). Map-based cloning of *alt1* revealed that *ALT1* encodes a Snf2 family chromatin remodeling ATPase. A functional analysis revealed that the alkaline tolerance of *alt1* is associated with reduced ROS accumulation under alkaline conditions (Guo et al., 2014). These findings suggest that, as for salt tolerance (Kao, 2017), alkaline tolerance in rice plants is correlated with ROS-scavenging capability.

This study aimed to gain insights into the mechanism of alkalinity-induced root damage by focusing on the effects that ROS have on rice seedlings. Our results strongly suggest that oxidative stress due to ROS accumulation damages root cells and inhibits the growth of rice seedlings grown under alkaline stress.

## Materials and Methods

### Plant Material and Growth Conditions

Two local rice cultivars with different levels of alkaline tolerance were used in this study: ‘Dongdao-4’ (D-4, moderately tolerant to SA) and ‘Jiudao-51’ (J-51, sensitive to SA) (Feng et al., 2016). D-4 is an elite cultivar in the local SA land area, which was bred by crossing ‘Akitakomachi’ with ‘Nongda-10’ in Da’an Sodic Land Experiment Station, Jilin, China (Yang et al., 2011). The cultivar J-51 was bred by crossing ‘Tong-35’ with ‘Nongda-3’ in Rice research institute, Jilin city Academy of Agriculture Science, Jilin, China (China Rice Data Center).

Seeds were surface-sterilized with 75% (v/v) alcohol for 5 min, and rinsed with deionized water five times. After immersing in water for 2 days, the seeds were sprinkled onto wet filter paper in a Petri dish, and germinated for 1 day at 28°C in a dark incubator. Eighteen uniformly germinated seeds were transplanted onto a multi-well plate floating on a 300-ml cup containing deionized water for 7 days, and then grown for 7 days in half-strength Kimura B nutrient solution (Miyake and Takahashi, 1983) in a controlled growth chamber under the following conditions: 25°C day/20°C night, 12-h photoperiod, and 350 μmol photons m^-2^ s^-1^ light intensity.

### Alkaline Treatment

We used Na_2_CO_3_ at 15 mM (pH = 10.87, EC = 2.7 mS/cm) to simulate alkaline stress. In our previous study, a series of Na2CO3 concentrations ranging from 0 to 50 mM was examined in correlation with cell injury and plant growth inhibition (Lv et al., 2013). Based on results of the study, we chose the lowest Na_2_CO_3_ concentration (15 mM) where a significant root damage and plant growth inhibition were observed during the experimental period (7 days).

Two-week-old rice seedlings were treated with or without 15 mM Na_2_CO_3_ solution for 3 days prior to all analyses.

### Pretreatment of Rice Seedlings with Procyanidins

The procyanidins are a group of polyphenolic compounds that exhibit high ROS-scavenging activity, and naturally occur in fruits, vegetables, nuts, seeds, flowers, and bark (Fine, 2000; Rue et al., 2017). Grape seed is a rich source of procyanidins, and grape seed extract (GSE) is widely used as an antioxidant dietary supplement. In this study, procyanidins (98% pure; Ci Yuan Biotechnology Co. Ltd., Shanxi, China) from GSE was used to assess the effect of ROS elimination on alkalinity-induced root damage and seedling growth. Two-week-old rice seedlings were pretreated for 24 h with 0.98% procyanidins prepared in water, followed by treatment with or without 15 mM Na_2_CO_3_ solution for 3 days.

### Measurement of Seedling Growth

After 3 days of Na_2_CO_3_ treatment, the survival rate of the rice seedlings was determined. Ten rice seedlings were randomly selected from the 25 seedlings in each treatment group and scanned using an Epson Expression 10000XL (Epson America Inc., Long Beach, CA, United States). The resulting images were digitized using the WinRHIZO program, according to the manufacturer’s instructions (Regent Instruments Canada Inc., Ville de Québec, QC, Canada), and the average shoot length (SL, the length of the longest leaf), total root length (TRL), total root surface area (RSA), root diameter (RD), total root volume (RV), and root number (RN) were determined. After scanning, the seedlings were cut and divided into shoots and roots, and their fresh weights measured. These samples were then dried in a forced-air-driven oven at 105°C for 2 h, followed by 70°C, until a stable mass was reached, prior to the determination of dry mass. The roots were rinsed four times with deionized water and then dried. Individual seedlings were classified as dead if all the leaves were dry and brown.

### Measurement of Membrane Injury (MI) and Malondialdehyde (MDA) Content

Membrane injury (MI) was measured by electrolyte leakage (Tantau and Dörffling, 1991). Ten seedlings were randomly selected from each treatment group, washed with deionized water to remove surface-adhered electrolytes, and cut and divided into shoots and roots. Samples (2 g fresh weight) of the shoots and roots were submerged in 15 ml of deionized water in 50-ml conical tubes and kept at 20°C for 1 h. The electrical conduction of the effusion was then measured (*R*1). The tissue samples were killed by heating tubes in a boiling bath for 40 min, cooled to 20°C, and the electrical conduction of the effusion was measured again (*R*2). The MI was evaluated using the formula MI (%) = *R*_1_/*R*_2_ × 100%.

Malondialdehyde (MDA) is a decomposition product of polyunsaturated fatty acid hydroperoxides, and is often used as an indicator of lipid peroxidation due to oxidative stress. The MDA content was determined by the thiobarbituric acid reaction as described by Heath and Packer (1968). A fresh root sample (0.1 g) was homogenized in 1 ml of 50 mM phosphate buffer (pH 7.8) using a bench-top ball-mill (Scientz-48, Ningbo Scientz Biotechnology Co. Ltd., Ningbo, China) at 50 Hz for 30 s, and centrifuged at 12,000 rpm for 15 min. Subsequently, 400 μl of supernatant was mixed with 1 ml of 0.5% thiobarbituric acid, and the mixture was placed in a boiling water bath for 20 min. The mixture was then cooled and centrifuged, and the absorbance of the resulting supernatant was measured at 532, 600, and 450 nm. The MDA content was calculated using the following formula: 6.45 × (A_532_ - A_600_) - 0.56 × A_450_.

### Measurement of Root Vigor and Cell Viability

Root α-naphthylamine-oxidizing activity was used as an indicator of root vigor according to the method described by Ramasamy et al. (1997). Rice roots (0.1 g) were harvested in a 10-ml tube containing 5 ml of 0.1 mol/l phosphate buffer (pH 7.0) and 25 μg/ml of α-naphthylamine and incubated at room temperature for 10 min. A 200-μl aliquot of the solution was then taken as the reaction control. The tube was further incubated at 25°C for 1 h, and a 200-μl aliquot of the solution was taken, mixed with 1 ml of distilled water, 100 μl of 1% sulfanilic acid anhydrous, and 100 μl of 1 mg/ml sodium nitrite, and incubated for 5 min. After diluting the mixture with 1.1 ml of distilled water, the resulting color was measured using a spectrophotometer at 510 nm. Root vigor is expressed as micrograms α-NA per gram fresh weight (FW) per hour (μg α-NA g^-1^ FW h^-1^).

Root cell viability was determined by Evan’s Blue staining (Romero-Puertas et al., 2004; Rodriguez-Serrano et al., 2006). Root tips (2 cm) were infiltrated with a 0.25% (w/v) aqueous solution of Evan’s Blue for 30 min, before being rinsed with deionized water until no further blue dye was eluted from the roots. The stained root tips were observed under a stereoscopic microscope (SMZ1270i, Nikon, Tokyo, Japan). To quantify the Evan’s Blue staining, five stained root tips were solubilized by incubating in 1% (w/v) SDS in 50% (v/v) methanol at 50°C for 30 min, and the absorbance was measured at 600 nm.

### Measurement of ROS Levels and Enzyme Activity

The H_2_O_2_ and $O_{2}^{•-}$ contents were measured using a H_2_O_2_ and $O_{2}^{•-}$ content determination kit, according to the manufacturer’s instructions (Comin Biotechnology, Suzhou, China).

For the determination of antioxidant enzyme activity, fresh roots (0.1 g) were loaded into a 2-ml tube and frozen in liquid nitrogen, then homogenized in 1 ml of 50 mM phosphate buffer (pH 7.8) using the bench-top ball-mill at 50 Hz for 30 s. The homogenate was centrifuged at 12,000 rpm for 15 min at 4°C, and the resulting supernatant was used for SOD, CAT, and POD assays.

Superoxide dismutase (EC 1.15.1.1) activity was determined using the nitro blue tetrazolium (NBT) method as described by Giannopolitis and Ries (1977). One unit of SOD was defined as the amount of enzyme required to cause 50% inhibition of NBT reduction, as monitored at 560 nm. CAT (EC 1.11.1.6) activity was measured according to the method described by Aebi (1984), and was assayed by the decline in absorbance per minute at 240 nm as a consequence of H_2_O_2_ consumption. POD (EC 1.11.1.7) activity was determined by assessing the rate of guaiacol oxidation in the presence of H_2_O_2_. One unit of POD was defined as the increase in absorbance per minute at 470 nm (Klapheck et al., 1990).

To assay APX activity, samples were extracted with 1 ml of 50 mM phosphate buffer (pH 7.0) containing 1 mM AsA and 1 mM ethylenediaminetetraacetic acid, and homogenized using the bench-top ball-mill at 50 Hz for 30 s. The homogenate was centrifuged at 12,000 rpm for 15 min at 4°C. Subsequently, the supernatant was mixed with phosphate buffer (pH 7.0), 15 mM AsA, and 0.3 mM H_2_O_2_. The reaction mixture was analyzed using a spectrophotometer at 290 nm. One unit of APX was defined as the variable quantity of absorbance per minute at 290 nm.

### Measurement of Chlorophyll Content

Leaf samples were extracted using a 5-ml mixture of ethanol and acetone (V:V = 1:1). The absorbance of the supernatant was determined at 645 and 663 nm. Total chlorophyll content was calculated using the following formula: 8.02OD_663nm_ + 20.21 OD_645nm_V/1000W.

### RNA Isolation and Quantitative Real-time Polymerase Chain Reaction (qRT-PCR)

Roots (0.2 g) were sampled in liquid nitrogen and ground using the bench-top ball-mill at 50 Hz for 30 s. Total RNA was extracted with TRIzol reagent (TaKaRa Bio, Tokyo, Japan), and first-strand cDNA was synthesized using M-MLV reverse transcriptase (Thermo Fisher Scientific, Carlsbad, CA, United States), according to the manufacturer’s protocols. A qRT-PCR was performed to determine the transcriptional expression of five genes related to cell death. Gene-specific primers were designed using Primer 5.0 software (**Table 1**). The housekeeping gene *β-actin* (GenBank ID: X15865.1) was used as an internal standard. The PCR was conducted in a 20-μl reaction mixture containing 1.6 μl of cDNA template (50 ng), 0.4 μl of 10 mM specific forward primer, 0.4 μl of 10 mM specific reverse primer, 10 μl of 2× SYBR^®^ *Premix Ex Taq* (TaKaRa Bio), and 7.6 μl of double-distilled H_2_O in a PCRmax machine (Eco TM 48, Illumina, Saffron Walden, United Kingdom). The procedure was performed as follows: 1 cycle for 30 s at 95°C, 40 cycles for 5 s at 95°C and 20 s at 60°C, and 1 cycle for 60 s at 95°C, 30 s at 55°C, and 30 s at 95°C for a melting curve analysis. The relative expression level was computed using the 2^-ΔΔC_T_^ method (Livak and Schmittgen, 2001).

**Table 1:** Primer pairs of cell death-related genes used for a quantitative real-time polymerase chain reaction.

Gene name	Primer sequences	Reference
(RAP-DB ID)	(5′→3′)	
*OsBI1*	CTACATCAAGCACGCACTC	Yang et al., 2012
(Os02g0125300)	ACCTCTTCTTCCTCTTCTTCTC	
*OsCP1*	TCAAGAACCAGGGCCAGTG	Yang et al., 2012
(Os04g0670500)	CCAGCTCCTGCTCCGACA	
*OsKOD1*	TCAAGCCATTCATCTTCCAT	Yang et al., 2012
(Os04g0507950)	ATCAGCAACCTCGTCAAG	
*OsHsr203j*	CGGCGAGGCTGAAGGAGAT	Yang et al., 2012
(Os01g0155000)	GATTCAGACCACATGAGAACACCA	
*OsNAC4*	TGGATGGAGCAAG×AAGG	Kaneda et al., 2009
(Os01g0816100)	CCACCACATTTGCAGAATCA	
*OsACT1*	TTCCAGCCTTCCTTCATA	Yu et al., 2015
(Os03g0718100)	AACGATGTTGCCATATAGAT	

### Experimental Design and Statistical Analyses

All of the experiments were conducted in a controlled growth chamber with three biological replicates, each consisting of five to ten plants. Statistical analyses were performed using the statistical software SPSS 21.0 (IBM Corp., Armonk, NY, United States). A one-way analysis of variance (ANOVA) was used to compare the treatment means (*P* = 0.05). SigmaPlot 10.0 (Systat Software Inc., San Jose, CA, United States) was used for graphical presentation of the data.

## Results

### Effect of Alkaline Stress on Seedling Growth

**FIGURE 1:**
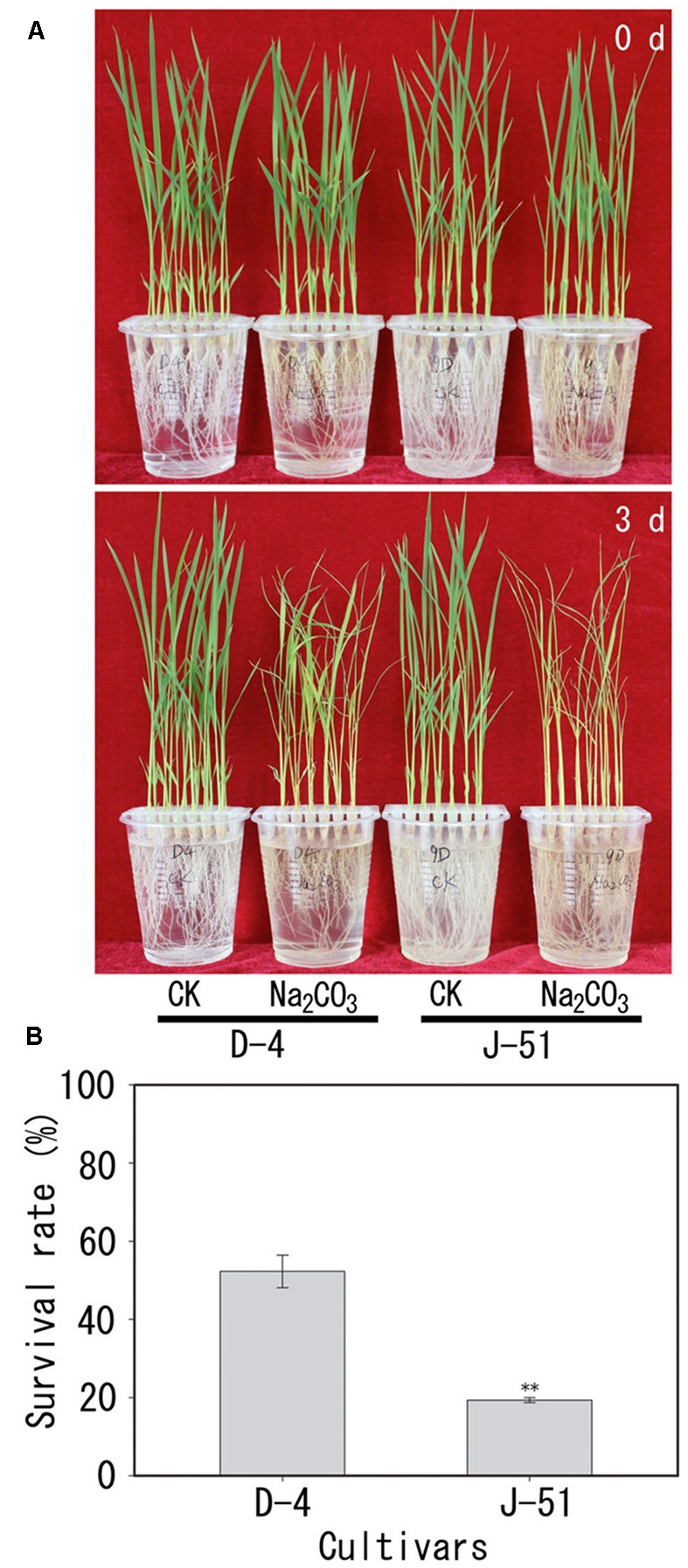
Images of growth **(A)** and survival rates **(B)** of rice seedlings under alkaline conditions. Two-week-old rice seedlings (0 days) were grown for 3 days under unstressed control (CK) or alkaline (Na_2_CO_3)_-stressed conditions.

**FIGURE 2:**
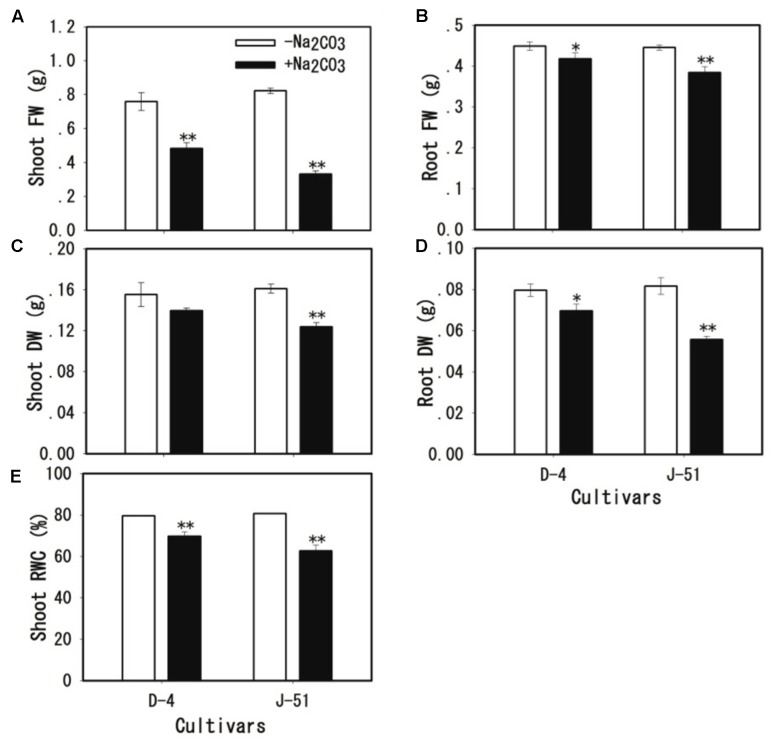
Fresh weight (FW) of shoots **(A)** and roots **(B)**, dry weight (DW) of shoots **(C)** and roots **(D)**, and relative water content (RWC) of shoots **(E)** of rice seedlings grown under unstressed control (open columns) or alkaline-stressed (filled columns) conditions for 3 days. Values are means ± SD, *n* = 3. Asterisks denote a significant difference to the control plants (^∗^*P* < 0.05, ^∗∗^*P* < 0.01).

**FIGURE 3:**
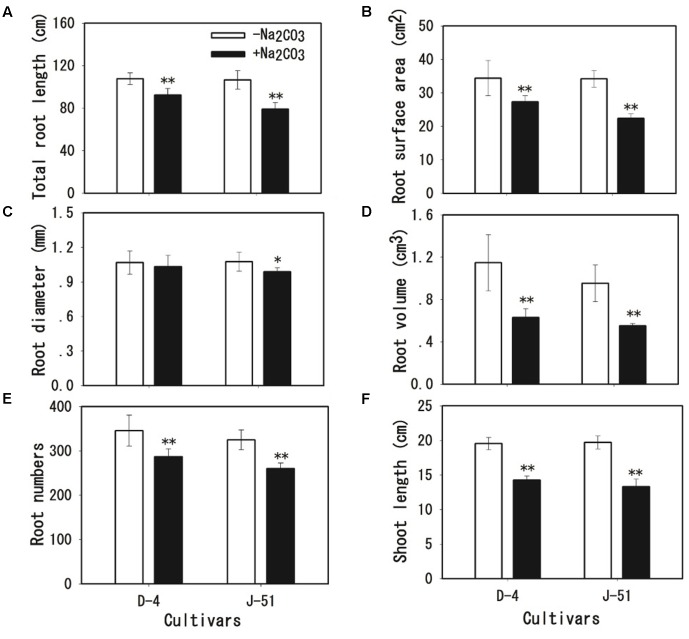
Shoot length **(A)**, total root length **(B)**, root surface area **(C)**, root diameter **(D)**, root volume **(E)**, and root number **(F)** of rice seedlings grown under unstressed control (open columns) or alkaline-stressed (filled columns) conditions for 3 days. Values are means ± SD, *n* = 3. Asterisks denote a significant difference to the control plants (^∗^*P* < 0.05, ^∗∗^*P* < 0.01).

Alkaline treatment for 3 days caused the wilting and death of seedlings of both cultivars (**Figure 1A**). The seedling survival rates under alkaline stress were reduced to 52.3 and 19.3% of the control treatment in D-4 and J-51, respectively (**Figure 1B**). Shoot FW, shoot dry weight (DW), shoot relative water content (RWC), root FW, and root DW were significantly reduced (**Figures 2A–E**). In addition, TRL, RSA, TRV, RN, and SL were significantly reduced in both cultivars as compared to the control treatment (**Figures 3A,B,D–F**). A smaller effect was observed on root diameter (**Figure 3C**). Overall, alkaline stress appeared to have more deleterious effects on the seedling growth of J-51 than that of D-4.

### Alkaline Stress-Induced Root Cell MI

**FIGURE 4:**
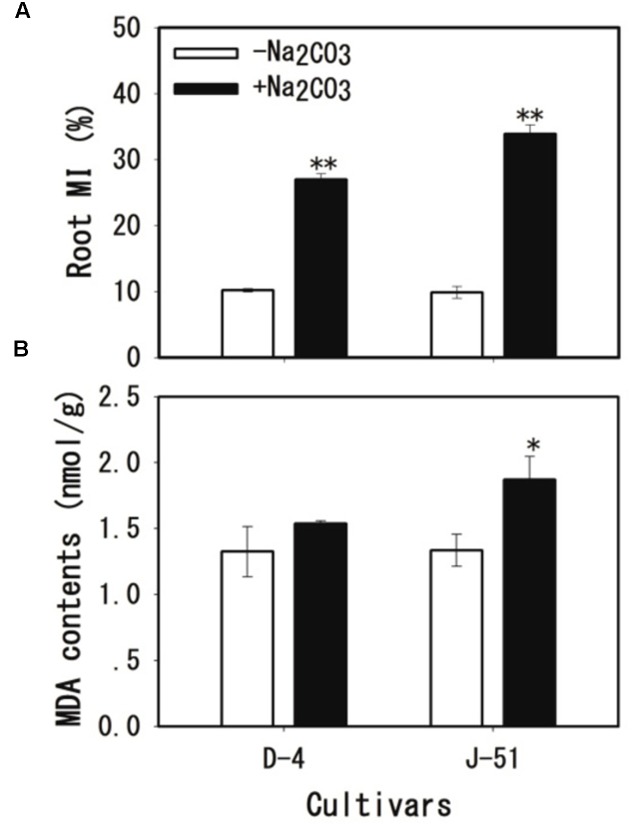
Membrane injury (MI) **(A)** and malondialdehyde (MDA) content **(B)** of rice roots grown under unstressed control (open columns) or alkaline-stressed (filled columns) conditions for 3 days. Values are means ± SD, *n* = 3. Asterisks denote a significant difference to the control plants (^∗^*P* < 0.05, ^∗∗^*P* < 0.01).

Root cell MI was assessed by the determination of MI (electrolyte leakage) and MDA content (lipid peroxidation). Alkaline treatment caused severe cell membrane damage in roots as shown by an increase in both MI (**Figure 4A**) and MDA content (**Figure 4B**), although the increase in MDA content in D-4 was not statistically significant (**Figure 4B**). MI and MDA induction under alkaline stress were higher in J-51 than in D-4.

### Alkaline Stress Decreased Root Cell Viability

**FIGURE 5:**
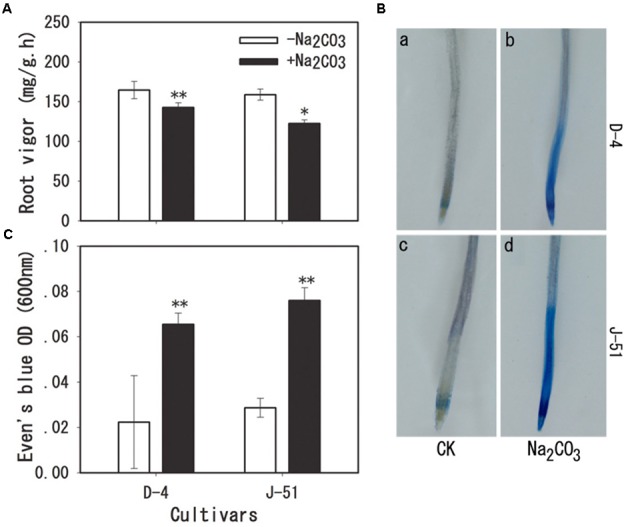
Root vigor **(A)** and cell viability **(B,C)** of rice roots grown under unstressed control (open columns) or alkaline-stressed (filled columns) conditions for 3 days. Cell viability was visualized by Evan’s Blue staining as described in the “Materials and Methods” section. Values are means ± SD, *n* = 3. Asterisks denote a significant difference to the control plants (^∗^*P* < 0.05, ^∗∗^*P* < 0.01).

Alkaline treatment markedly decreased root vigor in both cultivars as assessed by α-naphthylamine-oxidizing activity (**Figure 5A**), and the root vigor in J-51 was inhibited to a greater degree than that in D-4. In addition, strong Evan’s Blue staining of root tips was observed following alkaline treatment (**Figure 5B**). Measurement of Evan’s Blue levels extracted from the root tips showed a three- and two-fold increase in response to alkaline treatment in J-51 and D-4, respectively (**Figure 5C**). These results indicate severe root cell damage following alkaline stress.

### Induction of Cell Death-Related Genes under Alkaline Stress

**FIGURE 6:**
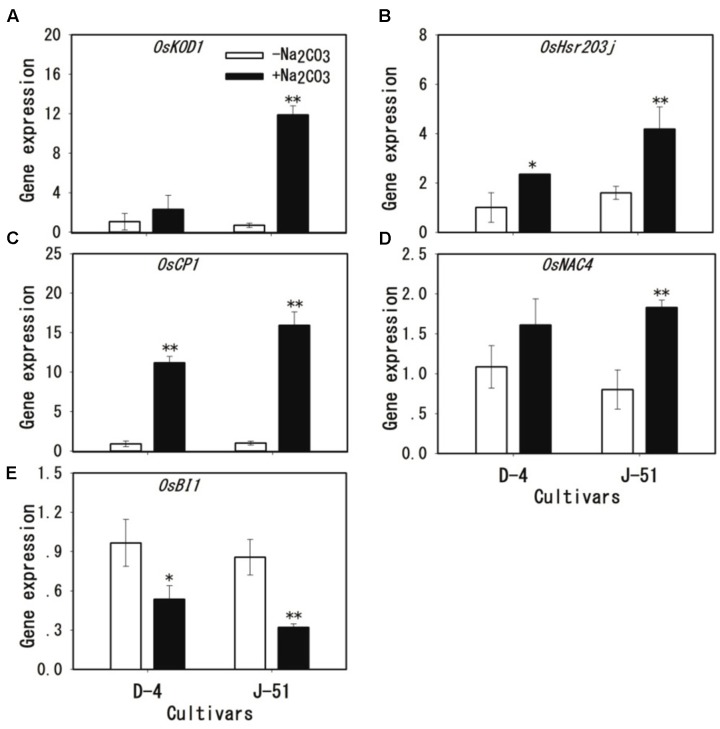
**(A–E)** Transcriptional induction of cell death-related genes under alkaline stress in rice roots. Two-week-old rice seedlings were grown under unstressed control (open columns) or alkaline-stressed (filled columns) conditions for 3 days. A quantitative real-time polymerase chain reaction was performed using *OsACT1* as an internal standard. Asterisks denote a significant difference to the control plants (^∗^*P* < 0.05, ^∗∗^*P* < 0.01).

To gain further insights into cellular damage in roots under alkaline stress, the transcription levels of five genes associated with cell death were analyzed: *OsKOD1*, an inducer of programmed cell death (PCD) (Blanvillain et al., 2011); *OsHsr203j*, which is often used as a cell death marker (Pontier et al., 1998; Wakasa et al., 2011); *OsCP1*, an executor of PCD (Lee et al., 2004; Li et al., 2011); *OsNAC4*, a NAC domain-containing transcription factor involved in cell death (Kaneda et al., 2009); and *OsBI1*, a cell death suppressor, which plays important roles in the modulation of PCD in response to abiotic and biotic stress (Weis et al., 2013). As shown in **Figure 6**, the expression of the cell death-associated genes *OsKOD1*, *OsHsr203j*, *OsCP1*, and *OsNAC4* was upregulated 12-, 4-, 16-, and 2-fold, respectively, in response to alkaline stress in J-51 (**Figures 6A–D**). Similarly, in D-4, these genes were all upregulated in response to alkaline stress (**Figures 6A–D**), although no statistical significance was observed for *OsKOD1* and *OsNAC4* (**Figures 6A,B**). Conversely, the cell death-suppressor gene *OsBI1* was significantly downregulated in both cultivars under alkaline stress.

### Alkaline Stress-Induced ROS Accumulation in Roots

**FIGURE 7:**
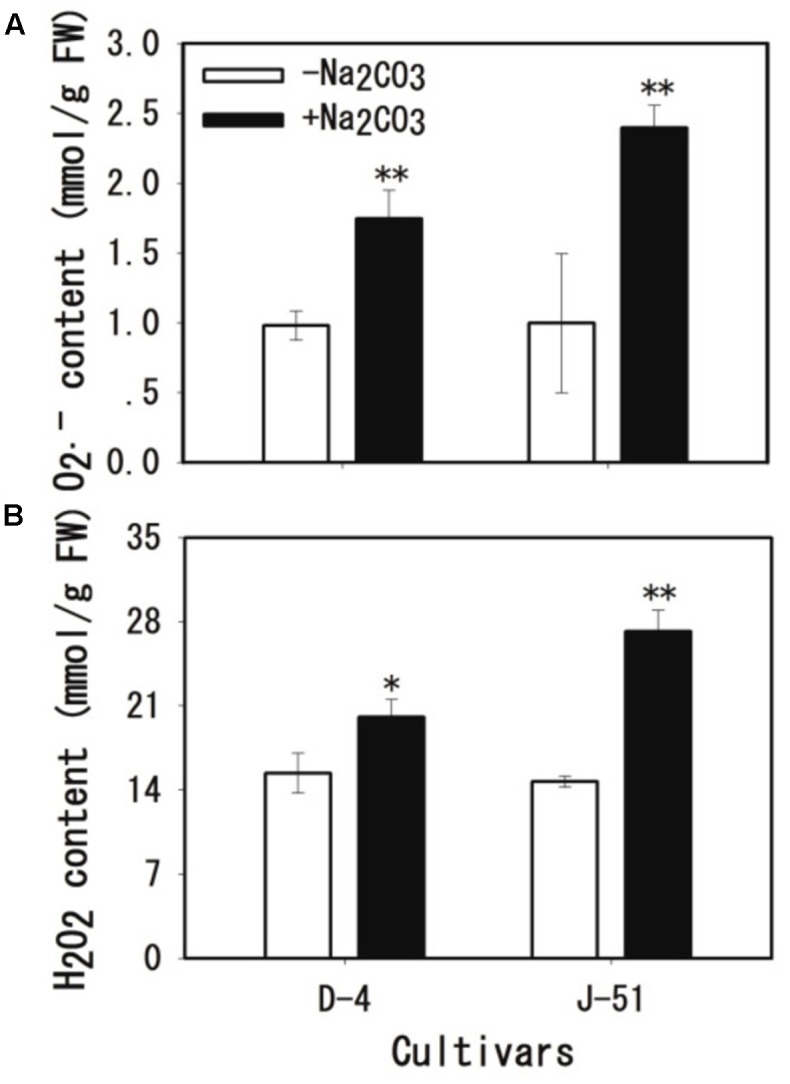
Increases in $O_{2}^{•-}$ **(A)** and H_2_O_2_ **(B)** levels in rice roots under alkaline stress. Two-week-old rice seedlings were grown under unstressed control (open columns) or alkaline-stressed (filled columns) conditions for 3 days. Values are means ± SD, *n* = 3. Asterisks denote a significant difference to the control plants (^∗^*P* < 0.05, ^∗∗^*P* < 0.01).

To investigate the possible mechanism of root damage under alkaline stress as shown in **Figures 4**–**6**, we determined the $O_{2}^{•-}$ and H_2_O_2_ contents. The results showed a remarkable accumulation of $O_{2}^{•-}$ (**Figure 7A**) and H_2_O_2_ (**Figure 7B**) in response to alkaline treatment in both cultivars. The levels of $O_{2}^{•-}$ and H_2_O_2_ accumulation under alkaline stress were much higher in J-51 than in D-4.

### Alkaline Stress Activated Antioxidant Enzymes

**FIGURE 8:**
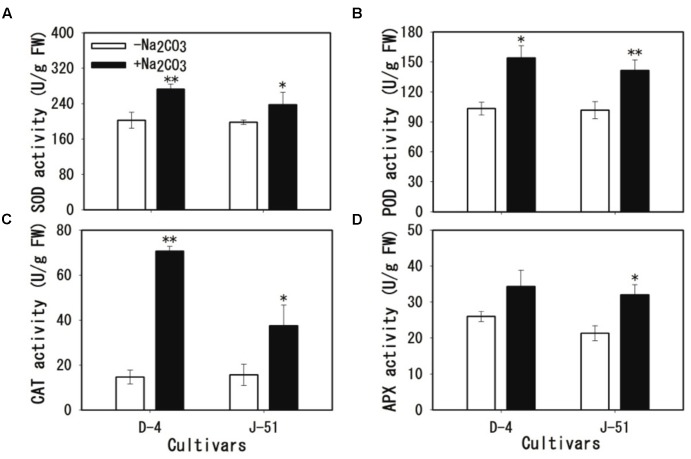
Activation of antioxidant enzymes in response to alkaline stress in rice roots. **(A)** Superoxide (SOD), **(B)** peroxidase (POD), **(C)** catalase (CAT), and **(D)** ascorbate peroxidase (APX). Two-week-old rice seedlings were grown under unstressed control (open columns) or alkaline-stressed (filled columns) conditions for 3 days. Asterisks denote a significant difference to the control plants (^∗^*P* < 0.05, ^∗∗^*P* < 0.01).

In response to stress-triggered ROS accumulation, the plants activated antioxidant defense mechanisms to eliminate excess ROS from their cells. As shown in **Figure 8**, the activity of the antioxidant enzymes SOD, POD, CAT, and APX were significantly increased in roots in response to alkaline stress; however, the increase in APX activity in D-4 was not statistically significant (**Figure 8D**). The activity of all of the antioxidant enzymes tended to be higher in D-4 than in J-51 plants.

### Exogenous Antioxidant (GSE) Mitigated Root Damage Caused by Alkaline Stress

**FIGURE 9:**
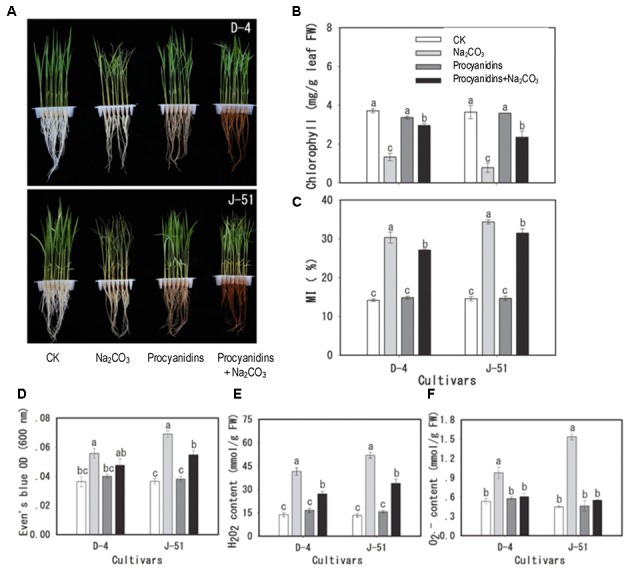
Mitigation of cellular damage by the exogenous antioxidant procyanidins under alkaline stress. Two-week-old rice seedlings were pretreated with or without 0.98% procyanidins for 24 h, then subjected to unstressed control (CK and procyanidins) or alkaline-stressed conditions (Na_2_CO_3_ and procyanidins+Na_2_CO_3_) for 3 days. **(A)** Images of seedling growth under different treatments, **(B)** chlorophyll content, **(C)** membrane injury (MI), **(D)** Evan’s Blue staining (cell viability), **(E)** H_2_O_2_, and **(F)** $O_{2}^{•-}$ content. The bars represent means ± SE, *n* = 3. The data were analyzed by conducting an analysis of variance, and different letters on the column denote a statistically significant difference (*P* < 0.05).

To investigate the association between root damage, the alkalinity-induced inhibition of plant growth (**Figures 1**–**6**), and ROS accumulation (**Figure 7**), we applied exogenous GSE that contained 98% natural procyanidins, which is a potent antioxidant. Exogenous GSE significantly reduced seedling wilt (**Figure 9A**), chlorophyll loss (**Figure 9B**), cell MI (**Figure 9C**), and cell death (**Figure 9D**) concomitantly with reduced ROS accumulation (**Figures 9E,F**) under alkaline stress.

## Discussion

High alkalinity (high pH) leads to deficiencies in nutritional minerals such as iron and phosphorus in the soil, and causes acute physiological stress to plants, which in turn, severely limits plant growth and agricultural productivity (Abrol et al., 1988; Qadir et al., 2006, 2007; Hossner, 2008; Wang et al., 2009; Amini et al., 2016). We previously reported that alkaline treatment markedly reduced rice seedling survival and total biomass, with the most serious adverse impact observed on root growth (Lv et al., 2013; Wei et al., 2015). It has been shown to cause severe damage to the root system, as demonstrated by a marked increase in cell injury and in the expression of the cell death-related gene *OsNAC4* (Lv et al., 2013; Wei et al., 2015). Consistently, Guo et al. (2014) showed that the alkaline-tolerant rice mutant *alt1* retained an intact root morphology under alkaline stress. The *alt1* mutant also accumulated significantly less H_2_O_2_ under alkaline conditions than a wild type control, suggesting a correlation between alkaline tolerance and a reduction in ROS accumulation in rice plants (Guo et al., 2014). In the present study, we showed that alkaline treatment increased MI (**Figure 4A**), MDA accumulation (**Figure 4B**), and Evan’s Blue staining (**Figure 5B**), and reduced root vigor (**Figure 5A**). It also upregulated the expression of the cell death-related genes *OsKOD1*, *OsHsr203j*, *OsCP1*, and *OsNAC4*, and downregulated the expression of a cell death-suppressor gene, *OsBI1* (**Figure 6**). Taken together, these results indicate that alkaline stress causes severe damage to root cells. Furthermore, we found that alkaline stress elicited marked ROS accumulation ($O_{2}^{•-}$ and H_2_O_2_) in the roots (**Figure 7**), and reducing the ROS accumulation by application of the antioxidant procyanidins to rice seedlings significantly alleviated alkalinity-induced root damage and increased seedling growth, concomitantly with a reduction in ROS accumulation (**Figure 9**). In addition, compared with the cultivar J-51 (alkaline-sensitive), D-4 (moderately alkaline-tolerant) exhibited lower levels of MI (**Figure 4A**) and MDA (**Figure 4B**), reduced expression of *OsKOD1*, *OsHsr203j*, and *OsNAC4* (**Figure 6**), and lower ROS accumulation (**Figure 7**). Our results strongly suggest that root cell damage, and thus growth inhibition of rice seedlings under alkaline stress, is closely associated with ROS accumulation. Excess ROS accumulation under various stressors is a major cause of cellular damage (Choudhury et al., 2017; Kao, 2017). It appears that the alkaline tolerance of rice plants is correlated with ROS-scavenging capability, as observed with salt tolerance (Kao, 2017). We previously reported that priming rice seedlings with abscisic acid (ABA) significantly mitigated damage to root cells and decreased seedling mortality and growth inhibition in response to alkaline stress, under both laboratory and field conditions (Wei et al., 2015, 2017). It would be interesting to determine whether the priming effect of ABA on alkaline tolerance is also associated with ROS homeostasis.

In response to ROS accumulation under various stressors, plants activate antioxidant defense systems to restore cellular ROS homeostasis by activating a set of antioxidant enzymes (Sewelam et al., 2016; Choudhury et al., 2017; Kao, 2017). In the present study, the antioxidant enzyme activity of SOD, POD, CAT, and APX were significantly increased in rice roots under alkaline stress (**Figure 8**). These data suggest that rice plants activate antioxidant defense systems in an attempt to cope with cellular damage caused by alkaline stress. However, the prolonged production of ROS under stress conditions may exceed the antioxidant defense capability, and result in oxidative damage to rice seedlings. In support of this hypothesis, reduced ROS accumulation following exogenous application of the antioxidant procyanidins significantly alleviated alkalinity-induced cellular damage and seedling growth (**Figure 9**). This suggests that increasing the ROS-scavenging capability of plants is critical for maintaining ROS homeostasis under alkaline stress, and would improve alkaline tolerance in rice plants. The transgenic overexpression of antioxidant enzyme genes has been shown to enhance ROS-scavenging activity and salt tolerance in rice plants (Tanaka et al., 1999; Zhao and Zhang, 2006; Motohashi et al., 2007; Kumar et al., 2014). Therefore, it is conceivable that strengthening ROS homeostasis by the molecular engineering of antioxidant enzyme gene(s) may help to improve alkaline tolerance and rice productivity in alkaline soils.

We previously reported a wide variation in alkaline tolerance among rice varieties (Lv et al., 2015; Feng et al., 2016). In the present study, we selected two rice cultivars with different tolerances to alkaline conditions, D-4 (moderately tolerant) and J-51 (highly sensitive) (Feng et al., 2016). These two cultivars exhibited marked differences in cellular damage and ROS accumulation in the roots: cultivar D-4 had lower levels of cellular damage (**Figures 4**–**6**) and ROS accumulation (**Figure 7**) than J-51. These results suggest that the alkaline tolerance of different cultivars is based, at least in part, on differences in ROS homeostasis. Moreover, D-4 had higher SOD, POD, and CAT activity than J-51 (**Figures 8A–C**), which may account for the lower levels of ROS accumulation observed under alkaline stress. Conversely, there was no significant activation of APX in D-4 under alkaline treatment (**Figure 8D**). These results indicate a difference in the recruitment of antioxidant enzymes for ROS homeostasis between different cultivars.

In summary, our results strongly suggest that ROS accumulation plays an important role in root cell damage and plant growth inhibition in rice seedlings under alkaline stress. These findings provide physiological insights into the molecular mechanisms of root damage in response to alkaline stress, and into molecular engineering for alkaline tolerance in rice.

## Author Contributions

Z-WL, C-JJ, and HZ designed the study; HZ, X-LL, H-YM, DL, R-XZ, H-YYu, H-YYa, and M-MW performed the laboratory experiments; HZ and X-LL performed the data collection, statistical analysis, and figure mapping; HZ and C-JJ wrote the manuscript; Z-WL provided scientific expertise.

## Conflict of Interest Statement

The authors declare that the research was conducted in the absence of any commercial or financial relationships that could be construed as a potential conflict of interest.

## References

[B1] Abrol I. Yadav J. S. P. Massoud F. (1988). *Salt-Affected Soils and Their Management.* Rome: Food & Agriculture Organization of the United Nations 10.1016/S0076-6879(84)05016-3

[B2] Aebi H. (1984). Catalase in vitro. *Method Enzymol.* 105 121–126. 10.1007/s11368-015-1293-1 6727660

[B3] Amini S. Ghadiri H. Chen C. Marschner P. (2016). Salt-affected soils, reclamation, carbon dynamics, and biochar: a review. *J. Soils Sediments* 16 939–953.

[B4] Amudha J. Balasubramani G. (2011). Recent molecular advances to combat abiotic stress tolerance in crop plants. *Biotechnol. Genet. Eng. Rev.* 6 31–58.

[B5] Blanvillain R. Young B. Cai Y. M. Hecht V. Varoquaux F. Delorme V. (2011). The Arabidopsis peptide kiss of death is an inducer of programmed cell death. *EMBO J.* 30 1173–1183. 10.1038/emboj.2011.14 21326210 PMC3061025

[B6] Caverzan A. Casassola A. Brammer S. P. (2016). “Reactive oxygen species and antioxidant enzymes involved in plant tolerance to stress,” in *Abiotic and Biotic Stress in Plants-Recent Advances and Future Perspectives*, ed. Shanker A. (Rijeka: InTech).

[B7] Chen S. Xing J. Lan H. (2012). Comparative effects of neutral salt and alkaline salt stress on seed germination, early seedling growth and physiological response of a halophyte species *Chenopodium glaucum*. *Afr. J. Biotechnol.* 11 9572–9581.

[B8] Choudhury F. K. Rivero R. M. Blumwald E. Mittler R. (2017). Reactive oxygen species, abiotic stress and stress combination. *Plant J.* 90 856–867. 10.1111/tpj.13299 27801967

[B9] Feng Z. H. Liu X. L. Jiang C. J. Liang Z. W. (2016). Comprehensive evaluation of rice (*Oryza sativa* japonica) germplasm for alkaline and saline tolerance at germination stage from Jilin province, China. *Soils Crops* 5 120–127. 10.11689/j.issn.2095-2961.02.009

[B10] Fine A. M. (2000). Oligomeric proanthocyanidin complexes: history, structure, and phytopharmaceutical applications. *Altern. Med. Rev.* 5 144–151. 10767669

[B11] Giannopolitis C. N. Ries S. K. (1977). Superoxide dismutases: I. Occurrence in higher plants. *Plant Physiol.* 59 309–314. 10.1104/pp.59.2.309 16659839 PMC542387

[B12] Gilliham M. Able J. A. Roy S. J. (2017). Translating knowledge about abiotic stress tolerance to breeding programmes. *Plant J.* 90 898–917. 10.1111/tpj.13456 27987327

[B13] Guo M. Wang R. Wang J. Hua K. Wang Y. Liu X. (2014). ALT1, a Snf2 family chromatin remodeling ATPase, negatively regulates alkaline tolerance through enhanced defense against oxidative stress in rice. *PLOS ONE* 9:e112515 10.1371/journal.pone.0112515 PMC4256374 25473841

[B14] Hanin M. Ebel C. Ngom M. Laplaze L. Masmoudi K. (2016). New insights on plant salt tolerance mechanisms and their potential use for breeding. *Front. Plant Sci.* 7:1787 10.3389/fpls.2016.01787 PMC5126725 27965692

[B15] Hartung W. Leport L. Ratcliffe R. G. Sauter A. Duda R. Turner N. C. (2002). Abscisic acid concentration, root pH and anatomy do not explain growth differences of chickpea (*Cicer arietinum* L.) and lupin (*Lupinus angustifolius* L.) on acid and alkaline soils. *Plant Soil* 240 191–199. 10.1023/A:1015831610452

[B16] Hazman M. Hause B. Eiche E. Nick P. Riemann M. (2015). Increased tolerance to salt stress in OPDA-deficient rice allene oxide cyclase mutants is linked to an increased ROS-scavenging activity. *J. Exp. Bot.* 66 3339–3352. 10.1093/jxb/erv142 25873666 PMC4449546

[B17] Heath R. L. Packer L. (1968). Photoperoxidation in isolated chloroplasts: I. Kinetics and stoichiometry of fatty acid peroxidation. *Arch. Biochem. Biophys.* 125 189–198. 10.1016/0003-9861(68)90654-1 5655425

[B18] Hong C. Y. Chao Y. Y. Yang M. Y. Cheng S. Y. Cho S. C. Kao C. H. (2009). NaCl-induced expression of glutathione reductase in roots of rice (*Oryza sativa* L.) seedlings is mediated through hydrogen peroxide but not abscisic acid. *Plant Soil* 320 103–115. 10.1007/s11104-008-9874-z

[B19] Hossner L. (2008). “Field pH,” in *Encyclopedia of Soil Science*, ed. Chesworth W. (Berlin: Springer), 271–272.

[B20] Islam M. S. Akhter M. El Sabagh A. Liu L. Y. Nguyen N. T. Ueda A. (2011). Comparative studies on growth and physiological responses to saline and alkaline stresses of Foxtail millet (*Setaria italica* L.) and Proso millet (*Panicum miliaceum* L.). *Aust. J. Crop Sci.* 5 1269–1277.

[B21] Kaneda T. Taga Y. Takai R. Iwano M. Matsui H. Takayama S. (2009). The transcription factor *OsNAC4* is a key positive regulator of plant hypersensitive cell death. *EMBO J.* 28 926–936. 10.1038/emboj.2009.39 19229294 PMC2670867

[B22] Kao C. H. (2017). Mechanisms of salt tolerance in rice plants: reactive oxygen species scavenging-systems. *Taiwan Nong Ye Yan Jiu* 66 1–8.

[B23] Klapheck S. Zimmer I. Cosse H. (1990). Scavenging of hydrogen peroxide in the endosperm of *Ricinus communis* by ascorbate peroxidase. *Plant Cell Physiol.* 31 1005–1013.

[B24] Kumar M. Lee S. C. Kim J. Y. Kim S. J. Kim S. R. (2014). Over-expression of dehydrin gene, *OsDhn1*, improves drought and salt stress tolerance through scavenging of reactive oxygen species in rice (*Oryza sativa* L.). *J. Plant Biol.* 57 383–393. 10.1007/s12374-014-0487-1

[B25] Lee D. H. Kim Y. S. Lee C. B. (2001). The inductive responses of the antioxidant enzymes by salt stress in the rice (*Oryza sativa* L.). *J. Plant Physiol.* 158 737–745. 10.1078/0176-1617-00174

[B26] Lee S. Jung K. H. An G. Chung Y. Y. (2004). Isolation and characterization of a rice cysteine protease gene, *OsCP1*, using T-DNA gene-trap system. *Plant Mol. Biol.* 54 755–765. 10.1023/b:plan.0000040904.15329.29 15356393

[B27] Li H. Yuan Z. Vizcay-Barrena G. Yang C. Liang W. Zong J. (2011). Persistent Tapetal *CELL1* encodes a PHD-finger protein that is required for tapetal cell death and pollen development in rice. *Plant Physiol.* 156 615–630. 10.1104/pp.111.175760 21515697 PMC3177263

[B28] Lin C. C. Kao C. H. (2001). Cell wall peroxidase activity, hydrogen peroxide level and NaCl-inhibited root growth of rice seedlings. *Plant Soil* 230 135–143. 10.1023/A:1004876712476 11164604

[B29] Livak K. J. Schmittgen T. D. (2001). Analysis of relative gene expression data using real-time quantitative PCR and the 2^-ΔΔC_T_^ method. *Methods* 25 402–408. 10.1006/meth.2001.1262 11846609

[B30] Lv B. S. Li X. W. Ma H. Y. Sun Y. Wei L. X. Jiang C. J. (2013). Differences in growth and physiology of rice in response to different saline-alkaline stress factors. *Agron. J* 105 1119–1128. 10.2134/agronj2013.0017

[B31] Lv B. S. Ma H. Y. Li X. W. Wei L. X. Lv H. Y. Yang H. Y. (2015). Proline accumulation is not correlated with saline-alkaline stress tolerance in rice seedlings. *Agron. J.* 107 51–60. 10.2134/agronj14.0327

[B32] Martinez-Beltran J. Manzur C. L. (2005). “Overview of salinity problems in the world and FAO strategies to address the problem,” in *Proceedings of the International Salinity Forum*, Riverside, CA, 311–313.

[B33] Mittler R. (2017). ROS are good. *Trends Plant Sci.* 22 11–19. 10.1016/j.tplants.2016.08.002 27666517

[B34] Miyake Y. Takahashi E. (1983). Effect of silicon on the growth of solution-cultured cucumber plant. *Soil Sci. Plant Nutr.* 29 71–83. 10.1080/00380768.1983.10432407

[B35] Moradi F. Ismail A. M. (2007). Responses of photosynthesis, chlorophyll fluorescence and ROS-scavenging systems to salt stress during seedling and reproductive stages in rice. *Ann. Bot.* 99 1161–1173. 10.1093/aob/mcm052 17428832 PMC3243573

[B36] Motohashi T. Nagamiya K. Prodhan S. H. Nakao K. Shishido T. Yamamoto Y. (2007). “Production of salt stress tolerant rice by overexpression of the catalase gene, *katE*, derived from *Escherichia coli*,” in *Proceedings Asia Pacific Conference on Plant Tissue and Agribiotechnology (APaCPA)*, Kuala Lumpur, 21.

[B37] Nor’aini M. F. Finch R. P. Burdon R. H. (1997). Salinity, oxidative stress and antioxidant responses in shoot cultures of rice. *J. Exp. Bot.* 48 325–331. 10.1093/jxb/48.2.325

[B38] Paz R. C. Rocco R. A. Reinoso H. Menéndez A. B. Pieckenstain F. L. Ruiz O. A. (2012). Comparative study of alkaline, saline, and mixed saline–alkaline stresses with regard to their effects on growth, nutrient accumulation, and root morphology of *Lotus tenuis*. *J. Plant Growth Regul.* 31 448–459. 10.1007/s00344-011-9254-4

[B39] Pontier D. Tronchet M. Rogowsky P. Lam E. Roby D. (1998). Activation of *hsr203*, a plant gene expressed during incompatible plant-pathogen interactions, is correlated with programmed cell death. *Mol. Plant Microbe Interact.* 11 544–554. 10.1094/mpmi.1998.11.6.544 9612953

[B40] Qadir M. Noble A. D. Schubert S. Thomas R. J. Arslan A. (2006). Sodicity-induced land degradation and its sustainable management: problems and prospects. *Land Degrad. Dev.* 17 661–676. 10.1002/ldr.751

[B41] Qadir M. Schubert S. Badia D. Sharma B. R. Qureshi A. S. Murtaza G. (2007). Amelioration and nutrient management strategies for sodic and alkali soils. *CAB Rev. Perspect. Agric. Vet. Sci. Nutr. Nat. Resour.* 21 1–13. 10.1079/PAVSNNR20072021

[B42] Qadir M. Schubert S. Ghafoor A. Murtaza G. (2001). Amelioration strategies for sodic soils: a review. *Land Degrad. Dev.* 12 357–386. 10.1002/ldr.458.abs

[B43] Radi A. A. Abdel-Wahab D. A. Hamada A. M. (2012). Evaluation of some bean lines tolerance to alkaline soil. *Eur. J. Biol. Res.* 2 B18–B27.

[B44] Ramasamy S. Ten Berge H. Purushothaman S. (1997). Yield formation in rice in response to drainage and nitrogen application. *Field Crops Res.* 51 65–82. 10.1016/S0378-4290(96)01039-8

[B45] Rodriguez-Serrano M. Romero-Puertas M. C. Zabalza A. Corpas F. J. Gomez M. Del Rio L. A. (2006). Cadmium effect on oxidative metabolism of pea (*Pisum sativum* L.) roots. Imaging of reactive oxygen species and nitric oxide accumulation in vivo. *Plant Cell Environ.* 29 1532–1544. 10.1111/j.1365-3040.2006.01531.x 16898016

[B46] Romero-Puertas M. C. Rodríguez-Serrano M. Corpas F. J. Gómez M. Río L. A. D. Sandalio L. M. (2004). Cadmium-induced subcellular accumulation of $O_{2}^{•-}$ and H_2_O_2_ in pea leaves. *Plant Cell Environ.* 27 1122–1134. 10.1111/j.1365-3040.2004.01217.x

[B47] Rue E. A. Rush M. D. van Breemen R. B. (2017). Procyanidins: a comprehensive review encompassing structure elucidation via mass spectrometry. *Phytochem. Rev.* 47 1–16. 10.1007/s11101-017-9507-3 PMC5891158 29651231

[B48] Sewelam N. Kazan K. Schenk P. M. (2016). Global plant stress signaling: reactive oxygen species at the cross-road. *Front. Plant Sci.* 7:187 10.3389/fpls.2016.00187 PMC4763064 26941757

[B49] Shasha C. (2012). Comparative effects of neutral salt and alkaline salt stress on seed germination, early seedling growth and physiological response of a halophyte species *Chenopodium glaucum*. *Afr. J. Biotechnol.* 11 9572–9581. 10.5897/ajb12.320

[B50] Singh A. K. Ansari M. W. Pareek A. Singla-Pareek S. L. (2008). Raising salinity tolerant rice: recent progress and future perspectives. *Physiol. Mol. Biol. Plants* 14 137–154. 10.1007/s12298-008-0013-3 23572881 PMC3550660

[B51] Tanaka Y. Hibino T. Hayashi Y. Tanaka A. Kishitani S. Takabe T. (1999). Salt tolerance of transgenic rice overexpressing yeast mitochondrial Mn-SOD in chloroplasts. *Plant Sci.* 148 131–138. 10.1016/S0168-9452(99)00133-8

[B52] Tantau H. Dörffling K. (1991). In vitro selection of hydroxyproline resistant cell lines of wheat (*Triticum aestivum*): accumulation of proline, decrease in osmotic potential, and increase in frost tolerance. *Physiol. Plant* 82 243–248. 10.1111/j.1399-3054.1991.tb00088.x

[B53] Tsai Y. C. Hong C. Y. Liu L. F. Kao C. H. (2004). Relative importance of Na+ and Cl– in NaCl-induced antioxidant systems in roots of rice seedlings. *Physiol. Plant* 122 86–94. 10.1111/j.1399-3054.2004.00387.x

[B54] Tsai Y. C. Hong C. Y. Liu L. F. Kao C. H. (2005). Expression of ascorbate peroxidase and glutathione reductase in roots of rice seedlings in response to NaCl and H_2_O_2_. *J. Plant Physiol.* 162 291–299. 10.1016/j.jplph.2004.06.004 15832681

[B55] Wakasa Y. Yasuda H. Oono Y. Kawakatsu T. Hirose S. Takahashi H. (2011). Expression of ER quality control-related genes in response to changes in *BiP1* levels in developing rice endosperm. *Plant J.* 65 675–689. 10.1111/j.1365-313X.2010.04453.x 21223397

[B56] Wang L. Seki K. Miyazaki T. Ishihama Y. (2009). The causes of soil alkalinization in the songnen plain of northeast China. *Paddy Water Environ.* 7 259–270. 10.1007/s10333-009-0166-x

[B57] Wei L. X. Lv B. S. Li X. W. Wang M. M. Ma H. Y. Yang H. Y. (2017). Priming of rice (*Oryza sativa* L.) seedlings with abscisic acid enhances seedling survival, plant growth, and grain yield in saline-alkaline paddy fields. *Field Crops Res.* 203 86–93. 10.1016/j.fcr.2016.12.024

[B58] Wei L. X. Lv B. S. Wang M. M. Ma H. Y. Yang H. Y. Liu X. L. (2015). Priming effect of abscisic acid on alkaline stress tolerance in rice (*Oryza sativa* L.) seedlings. *Plant Physiol. Biochem.* 90 50–57. 10.1016/j.plaphy.2015.03.002 25780993

[B59] Wei M. Y. Chao Y. Y. Kao C. H. (2013). NaCl-induced heme oxygenase in roots of rice seedlings is mediated through hydrogen peroxide. *Plant Growth Regul.* 69 209–214. 10.1007/s10725-012-9762-7

[B60] Weis C. Pfeilmeier S. Glawischnig E. Isono E. Pachl F. Hahne H. (2013). Co-immunoprecipitation-based identification of putative BAX INHIBITOR-1-interacting proteins involved in cell death regulation and plant-powdery mildew interactions. *Mol. Plant Pathol.* 14 791–802. 10.1111/mpp.12050 23782494 PMC6638788

[B61] Yamane K. Taniguchi M. Miyake H. (2012). Salinity-induced subcellular accumulation of H2O2 in leaves of rice. *Protoplasma* 249 301–308. 10.1007/s00709-011-0280-7 21533665

[B62] Yang C. Shi D. Wang D. (2008). Comparative effects of salt and alkali stresses on growth, osmotic adjustment and ionic balance of an alkali-resistant halophyte *Suaeda glauca* (*Bge*.). *Plant Growth Regul.* 56 179–190. 10.1007/s10725-008-9299-y

[B63] Yang F. Liang Z. W. Wang Z. C. (2011). Breeding and cultivation techniques of new rice variety of Dongdao 4. *Crops* 2 111 10.16035/j.issn.1001-7283.2011.02.027

[B64] Yang J. Zhao X. Cheng K. Du H. Ouyang Y. Chen J. (2012). A killer-protector system regulates both hybrid sterility and segregation distortion in rice. *Science* 337 1336–1339. 10.1126/science.1223702 22984070

[B65] Yu H. Jiang W. Liu Q. Zhang H. Piao M. Chen Z. (2015). Expression pattern and subcellular localization of the ovate protein family in rice. *PLOS ONE* 10:e0118966 10.1371/journal.pone.0118966 PMC4356581 25760462

[B66] Zhao F. Zhang H. (2006). Salt and paraquat stress tolerance results from co-expression of the *Suaeda salsa* glutathione S-transferase and catalase in transgenic rice. *Plant Cell Tissue Organ. Cult.* 86 349–358. 10.1007/s11240-006-9133-z

